# Obesity and Prader-Willi Syndrome Affect Heart Rate Recovery from Dynamic Resistance Exercise in Youth

**DOI:** 10.3390/diseases4010004

**Published:** 2016-01-15

**Authors:** Diobel M. Castner, Susan J. Clark, Daniel A. Judelson, Daniela A. Rubin

**Affiliations:** 1 Department of Kinesiology, California State University, Fullerton, 800 North State College Boulevard, Fullerton, CA 92834, USA; dan.judelson@nike.com (D.A.J.); drubin@fullerton.edu (D.A.R.); 2 Division of Pediatric Endocrinology, Children’s Hospital of Orange County, 1201 West La Veta Avenue, Orange, CA 92868, USA; sclark@choc.org

**Keywords:** adiposity, autonomic nervous system, heart rate recovery, childhood obesity, pediatric, physical activity

## Abstract

Following exercise, heart rate decline is initially driven by parasympathetic reactivation and later by sympathetic withdrawal. Obesity delays endurance exercise heart rate recovery (HRR) in both children and adults. Young people with Prader-Willi Syndrome (PWS), a congenital cause for obesity, have shown a slower 60-s endurance exercise HRR compared to lean and obese children, suggesting compromised regulation. This study further evaluated effects of obesity and PWS on resistance exercise HRR at 30 and 60 s in children. PWS (8–18 years) and lean and obese controls (8–11 years) completed a weighted step-up protocol (six sets x 10 reps per leg, separated by one-minute rest), standardized using participant stature and lean body mass. HRR was evaluated by calculated HRR value (HRRV = difference between HR at test termination and 30 (HRRV30) and 60 (HRRV60) s post-exercise). PWS and obese had a smaller HRRV30 than lean (*p* < 0.01 for both). Additionally, PWS had a smaller HRRV60 than lean and obese (*p* = 0.01 for both). Obesity appears to delay early parasympathetic reactivation, which occurs within 30 s following resistance exercise. However, the continued HRR delay at 60 s in PWS may be explained by either blunted parasympathetic nervous system reactivation, delayed sympathetic withdrawal and/or poor cardiovascular fitness.

## 1. Introduction

Increased body fat in youth is associated with various health conditions including altered autonomic nervous system (ANS) function and cardiovascular regulation [1], type II diabetes mellitus [2] and high overall risk for cardiovascular disease [3]. A population predisposed to early childhood morbid obesity includes a genetic disorder called Prader-Willi Syndrome (PWS), which has an incidence of 1 in 15,000 live births [4]. In addition to having distinct morphological features such as high fat mass, low lean mass and short stature, people with PWS present with hypotonia, poor muscle strength, low cardiovascular capacity and poor cardiovascular fitness [5,6,7,8]. Additionally, previous studies evaluating ANS dysregulation in PWS are equivocal and it remains unclear whether detriments are a result of parasympathetic [9,10] or sympathetic [1,11] inactivity.

Previous research has shown that overweight and obese young people experience lower resting vagal activity [12] and lower one-minute heart rate recovery (HRR) following maximal endurance exercise [13] compared to lean controls. HRR, an independent predictor of all-cause mortality in children [13] and adults [14], measures the HR decline from the end of exercise to a pre-determined time during recovery. Parasympathetic reactivation immediately occurs following exercise cessation [15] and is eventually replaced by sympathetic withdrawal at about 60 s into recovery [16]. Our previous investigation of 60-s HRR following endurance exercise suggested that HRR delay in children with PWS compared to lean and obese controls was attributed to either ANS dysregulation and/or poor cardiovascular fitness, both present in PWS individuals [1,5].

Endurance exercise is important, but recent works show that regular resistance exercise participation also has the potential to improve body composition and specific motor performance skills, increase spontaneous physical activity, and increase success and enjoyment in organized sport environments in young people with and without PWS [17,18,19]. Previous research has also shown that resistance training improves the HRR response following peak aerobic exercise in adults with [20] and without disability [21]. However, no studies have evaluated HRR immediately following an acute bout of dynamic resistance exercise in children or evaluated parasympathetic reactivation by measuring HRR at 30 s. Therefore, the purpose of this study was to investigate the effect of obesity and PWS on the HRR response at 30 and 60 s following a whole body resistance exercise protocol in children. The researchers hypothesized that resistance exercise HRR would be slower in those with increased adiposity compared to lean controls, with a greater delay observed in individuals with PWS, similar to the findings observed in the previous endurance exercise study [5].

## 2. Experimental Section

### 2.1. Recruitment and Participation Criteria

Eleven young people with PWS (ages 8 to 18 years), 16 obese (body fat percentage ≥ 95th percentile for age and sex, obese) and 12 lean (body fat percentage between the 2nd and 85th percentile for age and sex, lean) children without PWS (ages 8 to 11 years) participated in this study from July 2010 to August 2012 [22]. A larger age range for young people with PWS was implemented for participation because of the syndrome’s low prevalence and the lack of full sexual maturation in PWS [4], which places these young people at similar pubertal developmental stages as children without PWS. All PWS participants provided genetic testing documentation to confirm diagnosis: deletion (*n* = 10) and imprinting defect (*n* = 1). Young people with PWS also reported currently (*n* = 7), previously (*n* = 3) or never (*n* = 1) taking growth hormone replacement therapy. All participants and parents gave their informed assent and consent for inclusion before they participated in the study. The study was conducted in accordance with the Declaration of Helsinki, and the protocol was approved by the Institutional Review Boards from California State University, Fullerton (HSR# 13-0434), the Children’s Hospital of Orange County (IRB# 80308), and the United States Army Medical Research and Materiel Command (HRPO Log No. A-14648.1aii). A medical history questionnaire (completed by parents of participating children) and medical examination (young people with PWS only) determined participants with contraindications, excluding them from the study. Children with diabetes mellitus type 2, confirmed pregnancy or those unable to participate in moderate to vigorous physical activity were excluded from participation.

### 2.2. Procedures

Anthropometrics were measured with light clothing and no shoes using standardized procedures [23]. Body mass was measured via calibrated electronic scale (ES200L, Ohaus, Pinewood, NJ, USA) and stature via wall-mounted stadiometer (Seca, Ontario, CA, USA). Body mass index (BMI) was calculated by dividing body mass by stature in m^2^. BMI was used to calculate BMI z-scores. Body composition was measured using dual-energy X-ray absorptiometry following standard procedures (GE Healthcare, GE Lunar Corp., Madison, WI, USA). HR was measured via telemetry (Polar USA, Lake Success, NY, USA) and systolic (SBP) and diastolic (DBP) blood pressures were measured via standard sphygmomanometry (Silver Series DS65, Welch Allyn, Skaneateles Falls, NY, USA). Rating of perceived exertion (RPE) was recorded using the OMNI scale [24].

### 2.3. Dynamic Resistance Exercise Trial

Participants consumed a standardized breakfast (containing 260 kcal) two hours prior to visit arrival. After a five-minute walking or cycling warm-up participants completed the exercise trial. The dynamic resistance protocol consisted of six sets of 10 repetitions per leg of a step-up exercise onto an adjustable height platform while wearing a weighted vest [25,26]. One-minute rest periods separated each set. Relative work was standardized for all participants. For lean and obese, platform height was calculated as 20% of stature and vest load as 50% of lean body mass. To account for potential morphological or balance issues inherent to the syndrome, platform height was the same for all young people with PWS (23.0 cm); therefore, calculated vest load was modified such that relative work was equivalent among groups. For young people with PWS, the following calculation was used:

(1) Vest load in kg= [0.20(stature in cm) ×0.50(lean body mass in kg)] ÷23.0 cm

HR was measured at rest, throughout the exercise trial and for one minute during recovery (HRR). During the recovery period, participants were instructed to cease all activity and sit quietly and erect on the step. HRR was evaluated using calculated HRR value (HRRV). The value 30-s HRRV (HRRV30) was calculated as the difference between end of exercise HR and HR recorded after 30 s of exercise [15,16,27], while 60-s HRRV (HRRV60) was the difference after 60 s of exercise [27,28,29]. SBP and DBP were measured at rest, midway through and at the end of exercise. RPE was recorded at the end of each set. Time to complete the resistance trial was also recorded.

### 2.4. Statistical Analyses

**Figure 1:**
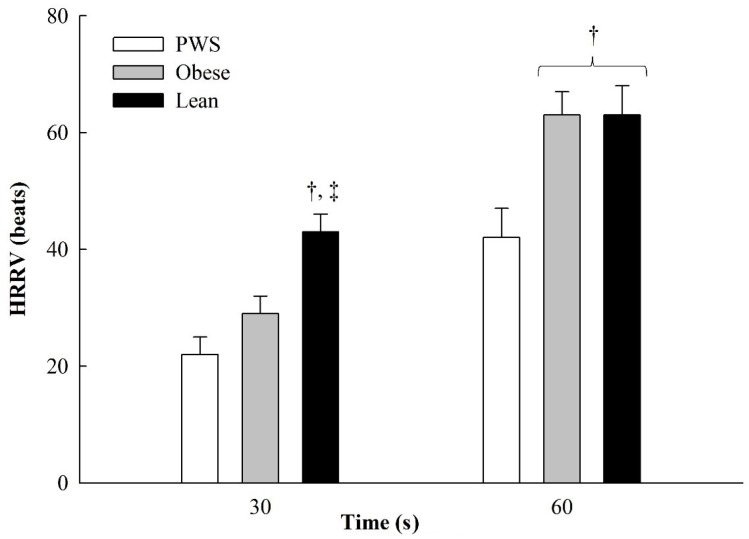
Heart rate 30 s and 60 s following dynamic resistance exercise in young people with Prader-Willi Syndrome (PWS) and lean and obese children without PWS, presented as mean + SE. ^†^ = different than PWS, ^‡^ = different than obese; *p* < 0.05.

Analyses were conducted using IBM SPSS Statistics 20 for Windows (SPSS, Inc., Chicago, IL, USA). One-way analysis of variance (ANOVA) tests were used to determine group differences for participant characteristics and all exercise responses. Due to the larger age range and varying usage of growth hormone replacement therapy in our group of young people with PWS, ANCOVAs for HRRV were initially ran controlling for these variables. However, these factors were not significant; thus, a three (group) by two (time) multivariate ANOVA was conducted to determine group differences for HRRV. Tukey’s post hoc tests were used to determine pairwise differences when group differences were significant. Significance level was set at *p* < 0.05 for all analyses. Sigma Plot for Windows version 10.0 (Systat Software, Inc., San Jose, CA, USA) was used to generate Figure 1.

## 3. Results and Discussion

### 3.1. Results

#### 3.1.1. Participant Characteristics and Responses to Dynamic Resistance Exercise

Participant characteristics are presented in Table 1. Young people with PWS were significantly older than lean (*p* = 0.02) and obese (*p* < 0.05). As expected, young people with PWS and obese presented with a significantly greater body mass, BMI, BMI z-score, body fat percentage and resting SBP compared to lean (*p* < 0.02 for all). Groups were similar for all other comparisons (see Table 1). Exercise responses are also presented in Table 1. Compared to lean and obese, young people with PWS had a lower platform height (*p* < 0.01 for both) and took longer to complete the exercise trial (*p* ≤ 0.01 for both). All other exercise responses were similar among groups with the exception of vest load, with young people with PWS carrying a heavier load than lean (*p* = 0.02). Additionally, lean presented a lower mid-exercise SBP than obese (*p* = 0.03).

**Table 1:** Participant characteristics and responses to dynamic resistance exercise by group, presented as frequency or mean (SE).

Variable	PWS (*n* = 11)	Obese (*n* = 16)	Lean (*n* = 12)
Sex (M/F)	5/6	11/5	7/5
Age (years)	11.4 (0.9)	9.5 (0.3) ^†^	9.1 (0.4) ^†^
Stature (cm)	144.6 (5.2)	144.0 (1.9)	139.5 (2.9)
Body mass (kg)	52.16 (5.70)	49.88 (2.37)	30.92 (1.92) ^†,‡^
BMI (kg∙m^−2^)	24.32 (1.24)	23.88 (0.75)	15.71 (0.38) ^†,‡^
BMI z-score	1.49 (0.24)	1.77 (0.13)	−0.53 (0.19) ^†,‡^
Body fat (%)	43.9 (2.3)	39.5 (1.1)	17.8 (1.5) ^†,‡^
Lean mass (kg)	27.53 (2.85)	28.67 (1.29)	24.05 (1.40)
Platform height (cm)	23.0	28.8 (0.4) ^†^	27.9 (0.6) ^†^
Vest load (kg)	17.90 (2.54)	14.33 (0.65)	12.02 (0.70) ^†^
Exercise duration (min)	17.0 (5.3)	12.3 (0.5) ^†^	12.2 (0.5) ^†^
HR (bpm)			
Resting	78 (3)	77 (2)	76 (4)
Exercise mean	153 (6)	165 (4)	152 (5)
End of exercise	162 (6)	176 (4)	160 (5)
SBP (mm Hg)			
Resting	107 (3)	99 (3)	85 (3) ^†,‡^
Mid-exercise	130 (5)	129 (5)	111 (4) ^‡^
End of exercise	123 (9)	128 (4)	114 (5)
DBP (mm Hg)			
Resting	64 (2)	62 (2)	57 (3)
Mid-exercise	82 (9)	70 (3)	69 (3)
End of exercise	76 (5)	63 (3)	67 (3)
RPE (OMNI)			
Exercise mean	5 (1.0)	6 (0.6)	5 (0.8)
End of exercise	6 (1.6)	7 (0.7)	6 (0.9)

^†^ = different than PWS. ^‡^ = different than obese. Significance level set at *p* < 0.05.

#### 3.1.2. Resistance Exercise HRR

HRRVs are presented in Figure 1. Despite young people with PWS being significantly older than lean and obese, age was not a significant covariate for HRRV. Young people with PWS and obese had a significantly lower HRRV30 than lean (*p* < 0.01 for both). In contrast, HRRV60 was significantly lower in young people with PWS than lean and obese people (*p* = 0.01 for both).

### 3.2. Discussion

The results of this study showed that excessive adiposity, with and without PWS, influenced HRR at 30 s but not 60 s. All groups responded similarly to the dynamic resistance exercise protocol, exhibited by similar exercise HR and end of exercise HR and BP. However, slower 30-s HRR in young people with PWS and obese children without PWS indicates that obesity delayed early parasympathetic reactivation. Conversely, obese children had a similar HRR as lean children at 60 s post-exercise while young people with PWS still presented a slower HRR than both groups of children without PWS. This latter finding suggests that other factors and not the excess adiposity may influence 60-s HRR in young people with PWS.

For this study in particular, delayed 30-s HRR in young people with PWS and obese children without PWS suggest that obesity delays early parasympathetic reactivation following exercise cessation. This finding is consistent with previous research in boys demonstrating that obesity altered reflex control during recovery from exercise [30]. Additionally, the present findings support the previously shown negative association between HRR and indices of obesity [31] and metabolic risk [32]. The relationship between obesity and blunted parasympathetic activity has been well documented in children [30,33,34,35]; however, the mechanisms that cause this dysfunction are a subject of debate. Lower than normal parasympathetic activity in obesity is argued to be caused by multiple organ systems [33], such as impaired baroreflex sensitivity [30], length-dependent peripheral ANS nerve dysfunction [33] and abnormal hypothalamus regulation [36]. Therefore, it is not surprising that parasympathetic reactivation from exercise in young people with PWS and obese controls was delayed.

A delayed HRR at 60 s may indicate a compromised response for parasympathetic reactivation, sympathetic withdrawal or both. In our study, excessive adiposity did not affect 60-s HRR as lean and obese children without PWS demonstrated the same response. Our previous study in submaximal endurance exercise generated similar findings [5]. We attributed the delay in HRR in young people with PWS to two potential factors: autonomic dysregulation and/or poor cardiovascular fitness. The parasympathetic system is the main regulator of the HR response to the exercise stimulus; initial parasympathetic withdrawal allows sympathetic activation to increase HR during exercise [37] and immediate parasympathetic reactivation drives HR decline during recovery from exercise [31]. In this study, young people with PWS had a similar resting and exercise HR as children without PWS, suggesting that vagal withdrawal and sympathetic activation during exercise were comparable to peers. However, the delayed HRR in young people with PWS at both 30 and 60 s indicate that both parasympathetic and sympathetic function during recovery may be compromised. Therefore, HRR in young people with PWS may be affected by compromised parasympathetic and sympathetic function inherent to the syndrome, as earlier hypothesized by previous researchers [1,9,10,11].

Another factor to consider when evaluating HR responses and regulation is absolute workload. Kingsley and colleagues (2014) showed that a heavier load during acute whole body resistance exercise induces decreased vagal activity and increased sympathetic drive during exercise in healthy adults [38]. Follow-up analyses revealed that young people with PWS (70.07 ± 8.11 kg) and children with obesity (64.21 ± 2.99 kg) had a similar absolute workload (*p* = 0.65) and workloads were greater than in lean (42.94 ± 2.59 kg; *p* < 0.01 for both). Thus, possibly the higher workload in obese and those with PWS could explain a delay in parasympathetic activation at 30 s. This, however, does not explain our results at 60-s HRR since obese children had a similar HRR as lean children and young people with PWS still exhibited a delayed response.

The delayed HRR at 60 s in young people with PWS may also be related with difficulty completing the resistance exercise protocol. In our study, young people with PWS took significantly longer to complete the exercise trial compared to peers without the syndrome (see Table 1), which may have resulted from physiological barriers such as decreased muscle strength (*i.e.*, hypotonia, muscle fiber size deficiency and atrophy) [8,39] and limited motor function (*i.e.*, lower motor proficiency) [40], both of which have been reported in PWS individuals. These factors could also contribute to poor cardiovascular fitness inherent to the syndrome. Young people with PWS have shown to participate in less spontaneous physical activity [41] and have a lower cardiovascular capacity than obese peers [5].

Cardiovascular fitness has been shown to influence HRR, as more fit individuals show a faster HRR than less fit individuals [20,21,42]. Thus, it is possible that poor cardiovascular fitness in young people with PWS also contributed to the compromised HR response following resistance exercise. Cardiovascular fitness may also explain similar HRRV60 in the control groups. Previous research has shown that cardiovascular fitness can increase parasympathetic activity in obese children, offsetting the otherwise blunted parasympathetic function typically seen in those with excessive adiposity [43,44]. The results of this study suggest that our group of obese children experienced delays in parasympathetic reactivation early in the recovery period (30-s HRR); however, were able to generate activity similar to that of lean children by 60 s. It is possible that our controls had similar cardiovascular fitness levels, although this is speculative since this factor was not measured directly.

In summary, multiple mechanisms may explain delayed HRR in young people with PWS. At 30-s HRR, increased adiposity or potentially prolonged sympathetic activation as a result of increased absolute workload seemed to delay parasympathetic reactivation in both young people with PWS and obese children without PWS. Additionally, slower 60-s HRR in young people with PWS compared to peers may potentially be attributed to diminished parasympathetic and sympathetic nervous system function inherent to the syndrome or lower cardiovascular fitness. This latter finding is further supported by the similar 60-s HRR found in lean and obese peers without the syndrome who may have had a similar cardiovascular fitness level.

This study had limitations in its design. First, HRR was measured for only one minute where parasympathetic and sympathetic functions likely overlap. Measuring HRR during the slow phase (*i.e.*, three to five minutes) will reveal potential detriments solely attributed to sympathetic withdrawal. Our choice was based on 60-s HRR being the most commonly measured value and one of the most reliable measurements of HRR [45]. Secondly, the resistance exercise protocol in this study is not one that is commonly used. However, the protocol created for this study allowed for us to safely investigate muscle strength in young people with PWS and has been shown to trigger hormonal and metabolic responses in both children (with and without PWS) [25] and adults [26]. Last, cardiovascular fitness was not measured and our groups may have had a wide range of cardiovascular fitness levels. Identifying participants with high cardiovascular fitness *versus* low could determine how much this factor influences HR following exercise independent from obesity. Future studies should consider discriminating between the role of cardiovascular fitness and obesity on HRR.

## 4. Conclusions

Following a dynamic resistance exercise protocol, 30-s HRR was reduced in participants with excessive body fat, suggesting delayed early parasympathetic reactivation. In contrast, 60-s HRR was slower only in young people diagnosed with PWS compared to lean and obese children without PWS, suggesting that altered parasympathetic and/or sympathetic function following exercise is due to syndrome-related factors and/or lower cardiovascular fitness. Future studies should examine HR for a longer duration (approximately three to five minutes post-exercise) to better describe sympathetic withdrawal following resistance exercise in children.
